# Early Enhancement of Neuroplasticity Index, the Ratio of Serum Brain-Derived Neurotrophic Factor Level to HAMD-24 Score, in Predicting the Long-Term Antidepressant Efficacy

**DOI:** 10.3389/fnbeh.2021.712445

**Published:** 2021-10-27

**Authors:** Yuxuan Zhang, Bo Cui, Tianyu Wang, Yan Lu, Zhenlin Chen, Zhilu Zou, Jinlin Miao, Xiuli Zhao, Yonggui Yuan, Haosen Wang, Gang Chen

**Affiliations:** ^1^Key Laboratory of Integrative Biomedicine for Brain Diseases, Center for Translational Systems Biology and Neuroscience, Nanjing University of Chinese Medicine, Nanjing, China; ^2^Interdisciplinary Institute for Personalized Medicine in Brain Disorders, Jinan University, Guangzhou, China; ^3^Department of Psychosomatics and Psychiatry, School of Medicine, Zhongda Hospital, Southeast University, Nanjing, China; ^4^School of Medicine, Institute of Psychosomatics, Southeast University, Nanjing, China; ^5^The Fourth People’s Hospital of Taizhou, Taizhou, China; ^6^Hubei University of Chinese Medicine, Wuhan, China; ^7^Co-innovation Center of Neuroregeneration, Nantong University, Nantong, China

**Keywords:** Yueju pill, BDNF, antidepressant efficacy, prediction, neuroplasticity index

## Abstract

**Background:** Current mainstream treatment of major depressive disorder (MDD) has a disadvantage in delayed onset of efficacy, making detection of early signatures predicative of the long-term treatment efficacy urgent.

**Methods:** MDD patients were scored with HAMD-24 and serum brain-derived neurotrophic factor (BDNF) levels were measured at different times in two independent trials: a single-arm observation of Yueju pill, a clinically approved traditional multiherbal medicine, and a two-arm random placebo-controlled trial for Yueju vs escitalopram. The ratio of the BDNF level to HAMD-24 score, or neuroplasticity index (NI), and its derived parameters were used for correlation analysis and receiver operating characteristic (ROC) analysis.

**Results:** On both the early (4th) and final (28th) days, Yueju and escitalopram significantly reduced HAMD-24 scores, compared to baselines, but only Yueju increased BDNF at both times. For either Yueju or escitalopram treatment, NI, but not BDNF, at baseline was correlated to NIs at the early or final treatment day. NI at early time was significantly correlated to early NI enhancement from the baseline for both Yueju and escitalopram, and to final NI enhancement from the baseline for Yueju in both trials. ROC analysis supported the predictability of Yueju’s final treatment efficacy from early NI enhancement.

**Limitations:** The small sample size and 28 days of treatment time may lead to the impossibility of ROC analysis of escitalopram.

**Conclusion:** Early NI enhancement is useful for prediction of long-term efficacy of Yueju and presumably some other antidepressants.

**Clinical Trial Registration:** [www.ClinicalTrials.gov], identifier [ChiCTR1900021114].

## Introduction

The major depressive disorder (MDD), or depression, is characterized by the loss of pleasure or interest in everyday activities, as well as other characteristics, including changes in sleep and appetite, low motivation, and suicidal ideation or action (Organization American Psychiatric, 2013; Otte et al., 2016). Current conventional antidepressants, mainly selective serotonin reuptake inhibitors (SSRIs), only have moderate rates of response and remission, with some unwanted side effects (Kogoj, 2014). Usually, long-term treatment period is required before the antidepressant effects of SSRIs can be elicited symptomatically (Agius and Bonnici, 2017). As depression is a progressive disease, the long lag period of the efficacy may lead to the miss of the optimal window for the intervention (Lane, 1998). Therefore, detection of early markers that can predict the treatment outcome is important for the improvement of the treatment. In the past decades, great efforts have been made to find the potential early biomarkers, including neurotropic factors, neurotransmitters, cytokines, as well as other molecules in the blood (Nutt, 2008; Dowlati et al., 2010; Tavakolizadeh et al., 2018; Xu et al., 2019). However, up to date, none of the individual predictors is robust enough to guide first-line treatment options (Kessler, 2018).

Brain-derived neurotrophic factor (BDNF) is a neurotrophic factor critical for the viability of neurons in neural circuit (Monteggia et al., 2007). A number of studies have shown that BDNF is profoundly implicated in depression (Peng et al., 2018; Koo et al., 2019; Szuhany and Otto, 2020). Studies have shown that BDNF levels are lower in depression patients than healthy people (Karege et al., 2002). Decreasing BDNF expression leads to deficient neural plasticity in the brain of patients with MDD, which makes it a potential biomarker to monitor these diseases (Peng et al., 2018). BDNF in serum or plasma is increasingly used as a putative biomarker for depression, and some studies revealed that lower levels of BDNF in the blood of depressed patients are improved after long-term treatment of certain antidepressants (Shimizu et al., 2003; Gazal et al., 2012; Vanicek et al., 2019). However, a considerable number of studies demonstrated no effect or even opposite findings (Kheirouri et al., 2016; Ryan et al., 2018). Therefore, whether the changes of blood BDNF levels at the early time of antidepressants treatment alone predict the long-term treatment efficacy remains elusive (Kessler, 2018; Xu et al., 2019).

Traditional medicine has been used as an alternative monotherapy or an adjunct to conventional antidepressant therapy, whose efficacy and safety has been supported on the basis of increasing number of clinical trials (Zhang et al., 2007; Qin et al., 2011; Wang et al., 2012; Yang et al., 2020). Yueju pill is a multiherbal pill formulated 800 years ago to treat syndromes associated with mood disorders (Ren and Chen, 2017; Wang et al., 2019). It contains multiple antidepressant components and some of the bioactive compounds have been identified (Wei et al., 2008; Zhang et al., 2015; Wu et al., 2016). More recently, a number of preclinical studies using various animal models demonstrated that a single high dose of Yueju was capable to elicit antidepressant activity in a fast-onset and persistent manner (Xue et al., 2013), hippocampus is one of many regions that are involved in depression-related behavior (Ren et al., 2016; Xia et al., 2016). A pilot perspective random controlled clinic trial showed that the use of high dose of Yueju contributed to the early onset of the alleviation of depressive symptoms, starting at 4 days after treatment together with fluoxetine (Wu et al., 2015). Additionally, chronic treatment of conventional low dose of Yueju pill also elicited antidepressant activity, with overall superior efficacy to fluoxetine, in an animal model of depression (Zou et al., 2017). Both single high dose and repeated low dose of Yueju pill significantly induce hippocampal BDNF synthesis through transcriptional and translational mechanisms (Xue et al., 2013).

Here, in a clinical observation of the effect of low dose of Yueju pill treatment, we disclosed that the ratio of serum BDNF level to the scale of depression using the scores of HAMD-24, defined as neuroplasticity index (NI), may be useful for prediction of the antidepressant treatment outcome. We thus carried out a prospective random placebo-controlled clinical trial, in which the monotherapy of Yueju pill was compared with the mainstream antidepressant escitalopram. We assessed the correlation of baseline NI with the changes of NI at different treatment times. The results indicated significant correlations of NI or their changes at different times post treatment of Yueju as well as escitalopram. Additionally, the change of NI at the early time may be used for prediction of the long-term antidepressant efficacy.

## Materials and Methods

### Experimental Design

**FIGURE 1:**
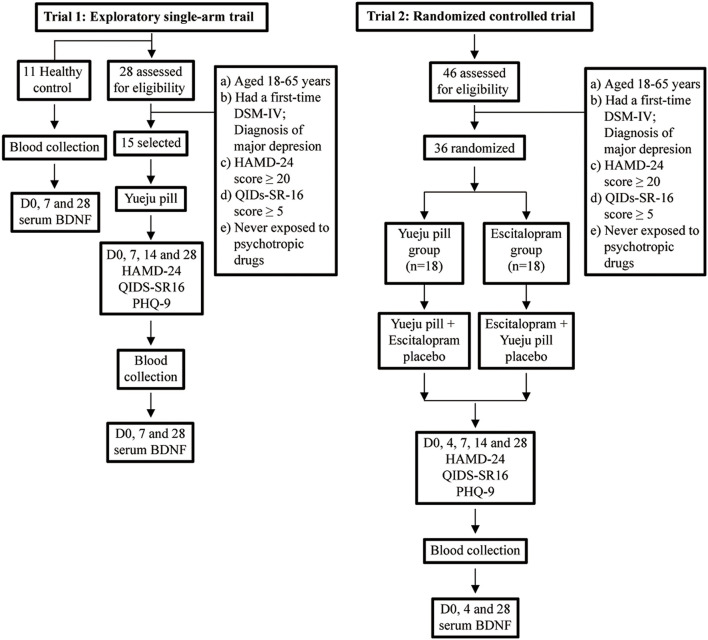
Flowchart of enrollment, randomization, discontinuation, and completion of the treatment phases. HAMD-24, QIDS-SR16, and PHQ-9 scores and serum BDNF level were collected as indicated.

This study consisted of two trials and was approved by the Institutional Review Board of The Fourth People’s Hospital of Taizhou (Figure 1). The first trial was an open-labeled exploratory single-arm trial. Patients (HAMD-24 scores (20) were recruited (Addington et al., 1996) and treated with the conventional dose of Yueju pill. The second trial was a double-blinded, random and placebo-controlled parallel one. Patients were diagnosed with MDD, and randomly assigned to Yueju pill and escitalopram treatment groups. The procedures were conducted according to institutional guidelines, and all participants have provided written informed consent (Chinese Clinical Trial Registry: ChiCTR1900021114).

### Trial 1: Exploratory Single-Arm Trial

The patients, who were consecutive recruited from August, 2016 to December, 2016, were diagnosed according to the DSM-IV of MDD (Table 1). The enrollment criteria were as follow: (1) 18–65 years old, (2) no psychotic characteristics but with diagnostic criteria for depression (DSM-IV), (3) no psychotropic medications previously, (4) HAMD-24 scores ≥20, (5) QIDS-SR16 scores ≥5, (6) never exposed to psychotropic drugs, (7) have not received antidepressant treatment within 1 week.

**TABLE 1:** Demographic data of the enrolled subjects with MDD or healthy controls.

Trials	Groups	Number	Male	Female	Age (years)
Trial 1 Open labeled	Yueju pill	15	1	14	42.93 ± 3.21
	Healthy control	11	6	5	41.7 ± 3.06
Trial 2 Double blinded	Yueju pill	18	5	13	45.17 ± 3.26
	Escitalopram	18	5	13	43.39 ± 3.51

#### Treatments and Measurements

The medicinal plants used to prepare Yueju are *Cyperus rotundus* L. (Xiang Fu), *Ligusticum chuanxiong* Hort (Chuan Xiong), *Gardenia jasminoides* J. Ellis. (Zhi Zi), *Atractylodes lancea* (Thunb.) DC. (Cang Zhu), and *Massa Fermentata* (Shen Qu) (Ren and Chen, 2017). Patients who met the enrollment criteria were enrolled and treated with Yueju pills (12 g/day, Jiangsu 707 Natural Pharmaceutical Co., Ltd., approval number, Z32020738). On Day 7 (D7), D14, and D28, scales of depression were assessed with the HAMD-24, which was scored by a practicing physician, as well as QIDS-SR16 and PHQ-9, which were scored by the patients themselves. On the baseline day D0, as well as post treatment days of D7 and D28, peripheral blood was drawn for BDNF measurements. The peripheral blood samples were also collected from healthy counterpart controls.

### Trial 2: Randomized Controlled Trial

Patients were consecutive recruited from March, 2017 to January, 2018, were diagnosed according to the DSM-IV of MDD (Table 1). The enrollment criteria were as follow: (1) 18–65 years old, (2) no psychotic characteristics but with diagnostic criteria for depression (DSM-IV), (3) no psychotropic medications previously, (4) HAMD-24 scores ≥20, (5) QIDS-SR16 scores ≥5, (6) never exposed to psychotropic drugs, (7) have not received antidepressant treatment within 1 week, (8) emmetropia and right hand. Patients who met the enrollment criteria were randomly assigned into two groups according to the enrollment order. The patients in the experimental group were treated with Yueju pills and the placebo for escitalopram. The patients in the escitalopram group were treated with escitalopram and herbal placebo. The placebo was prepared to be identical to the Yueju pill or escitalopram in shape, size, and color. The drugs and placebo are placed in the same packaging and the same box.

#### Treatments and Measurements

*Yueju group*: To accelerate the antidepressant response and then maintain the antidepressant activity, the patients took a high dose (23 g/time/day) of Yueju during the first week, followed by the dose of 12 g/time/day of Yueju from the second to fourth week. *Escitalopram group*: Escitalopram was made by Yamagata Pharma Co., Ltd. (National Medicine Standard H20080599). Patients took the escitalopram or corresponding placebo 10 mg twice a day, whereas Yueju pills and the herbal placebo were taken once a day after dinner. HAMD-24, QIDS-SR16, and PHQ-9 scales were scored on D0, D4, D7, D14, and D28. Peripheral blood was drawn for BDNF level testing on D0, D4, and D28.

### Blood Samples and Brain-Derived Neurotrophic Factor Measurements

Peripheral blood extractions in patients or healthy controls were performed between 9 and 10 am to minimize the effects of possible circadian rhythm alterations and the blood samples were collected into coagulation-promoting tubes. Patients were not exposed to drugs known to affect the coagulation system within 10 days prior to blood extraction. The blood samples were centrifuged (1,000 × *g* for 20 min) immediately and serum samples were collected and stored at −80°C until further analysis. BDNF level was measured using an anti-BDNF enzyme-linked immunosorbent assay (ELISA) kit (Boster Biological Technology Co., Ltd.) according to the manufacturer’s instructions. Diluted serum (1:20) with sample buffer was used to conduct BDNF ELISA in duplicate. BDNF standard solution was diluted to concentrations from 0 to 4,000 pg/ml to create the standard curve. Four patients in each group of Trial 2 failed to take the blood for this test.

### Statistics Analysis

All statistical analyses were conducted with SPSS 19.0 statistical software (Statistical Package for the Social Sciences, SPSS. Inc., Chicago, IL, United States), GraphPad Prism 6 (GraphPad Software, San Diego, CA, United States) and MedCalc statistical software (MedCalc Software Ltd., Ostend, Belgium). Analyses of variance with repeated measures as well as Pearson’s correlations were used as appropriate. Pearson’s correlations were performed with the raw values. For the receiver operating characteristic (ROC) analysis, HAMD-24 reduction rate (60% within 4 weeks) was defined to be quasi-effective. ROC curves were drawn to evaluate the prediction of the data collected in this study. Significance was evaluated at *p* < 0.05, two tailed. Data are reported as means ± SE.

## Results

### Both Yueju Pill and Escitalopram Demonstrated Early Improvement and Continuous Antidepressant Effects

**FIGURE 2:**
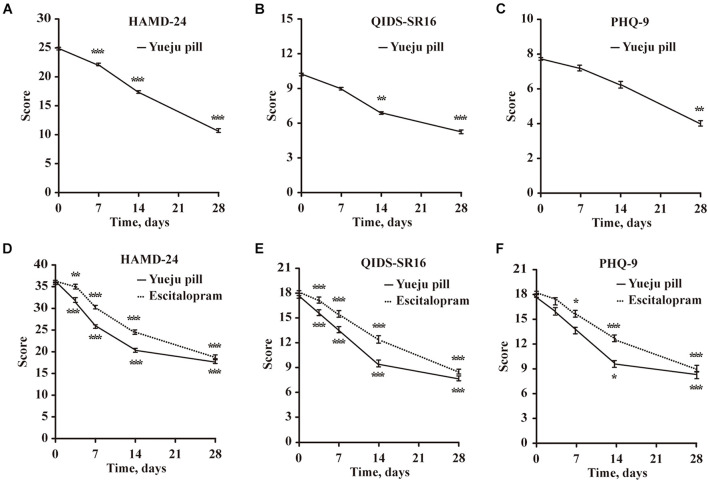
Time course changes of depression scores measured with HAMD-24, QIDS-SR16, and PHQ-9 after treatment in Trial 1 and Trial 2. **(A–C)** Depression scores Trial 1 in a single-arm trial, with low dose of Yueju Pill treatment for 4 weeks (*n* = 15). **(D–F)** Depression scores in Trial 2 a two-arm randomized controlled trial, with Yueju Pill (*n* = 18) or escitalopram treatment (*n* = 18) for 4 weeks. Values are expressed as means and standard errors. *p < 0.05, **p < 0.01, ***p < 0.001, compared with the baseline.

In Trial 1, 15 MDD patients were treated with low dose of Yueju pill for 4 weeks. Based on the analysis of HAMD-24 scores, there was a significant effect on time [*F*(1.377,9.28) = 77.51, *p* < 0.001, Figure 2A]. *Post hoc* tests indicated a trend of improvement of D7, and the significant improvement from D14 to D28 of treatment, compared to D0 (D7, *p* = 0.116; D14, *p* < 0.001, and D28, *p* < 0.001). The analysis of QIDS-SR16 [*F*(1.534,21.48) = 6.83, *p* < 0.001] showed the similar results, with a significant improvement from D14 to D28 (D7, *p* = 0.152, D14, *p* = 0.002, and D28, *p* < 0.001) (Figure 2B). For PHQ-9 [*F*(1.699,23.78) = 26.63, *p* < 0.001], the significant improvement was only on D28 (D7, *p* = 0.653, D14, *p* = 0.211, and D28, *p* < 0.001) (Figure 2C). The results from this observation demonstrated an antidepressant efficacy, with a plausible early symptomatic improvement.

In Trial 2, 36 patients were randomly assigned into placebo-controlled groups of escitalopram or Yueju (with high dose for the first week, followed with low dose for 3 weeks). For HAMD-24 scores, there was a significant effect for the interaction between drug and time [*F*(4,68) = 2.854, *p* = 0.03] and for time only [*F*(4,68) = 148.8, *p* < 0.001], but not for treatment only [*F*(1,17) = 0.507, *p* = 0.486]. Patients in Yueju group showed significant improvement at all times from D4 to D28, in comparison to D0 (*p* < 0.001 for D4, D7, D14, and D28, respectively). Similarly, escitalopram group showed significant improvement at all different time points (D4, *p* = 0.013; *p* < 0.001 for D7, D14, and D28, respectively). QIDS-SR16 scores started to improve from the fourth day and continued to the 28th day (Figures 2D,E). For PHQ-9 scores, Yueju group showed significant improvement at Day 7 (Figure 2F), whereas the escitalopram group started it at Day 14 (Figure 2F). These data indicate that escitalopram and initial high dose of Yueju elicited early antidepressant efficacy, and the significant improvement within the first 4 weeks were appreciable for both Yueju and escitalopram.

### The Serum Brain-Derived Neurotrophic Factor Levels at the Early and Final Treatment Time Were Improved in Yueju but Not Escitalopram Group

**FIGURE 3:**
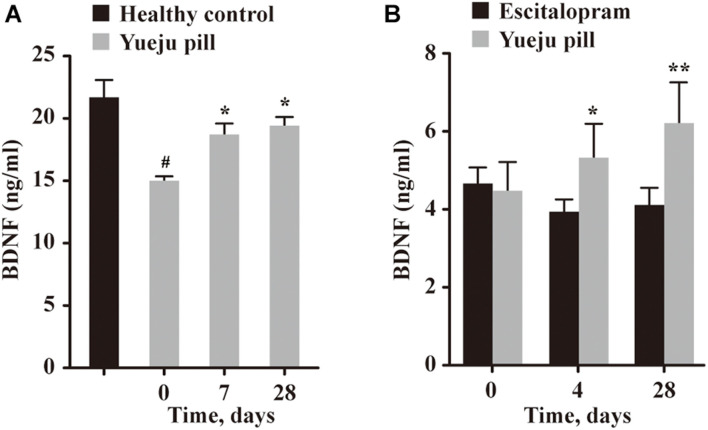
Serum BDNF levels at different time after treatment. **(A)** BDNF levels in healthy control group and at different times in depressive patients treated with Yueju pill in Trial 1 (*n* = 14). **(B)** BDNF levels at different times in depressive patients treated with Yueju pill (*n* = 14) or escitalopram (*n* = 14) in Trial 2. *p < 0.05, **p < 0.01, compared with the baseline **(A,B)**; ^#^*p* < 0.05, compared with healthy control **(A)**.

In Trial 1, serum BDNF level before treatment of 15 selected patients is much lower than the healthy controls (Figure 3A). The BDNF levels showed a significant effect on time [*F*(14,28) = 6.352, *p* < 0.001]. *Post hoc* tests showed a significant improvement on day 7 and day 28 (D7, *p* = 0.042 and D28, *p* = 0.023), compared with D0, and BDNF levels for both D7 and D28 after Yueju treatment were comparable to healthy controls (*p* > 0.05) (Figure 3A).

In Trial 2, there was a significant interaction between treatment and time [*F*(2,26) = 8.234, *p* = 0.002], but not for time alone [*F*(2,26) = 1.801, *p* = 0.185] or drug alone [*F*(1,13) = 0.729, *p* = 0.409]. Only Yueju group showed significant changes overtime [*F*(13,26) = 7.192, *p* = 0.0037], and there was a significant improvement on D4, *p* = 0.037 and on D28, *p* = 0.004. In escitalopram group, the BDNF levels did not have a significant change overtime on time [*F*(13,26) = 1.232, *p* = 0.3056] (Figure 3B).

### Correlation Analysis of Neuroplasticity Index and Neuroplasticity Index Enhancement

No significant correlation between serum BDNF levels with the degree of depression was found when the data from the two trials were analyzed separately. When the data in were collapsed, the baseline serum BDNF levels and HAMD-24 scores were found to be negatively correlated (*R*^2^ = 0.21, *p* = 0.002). Additionally, analysis of collapsed data from two trials indicated serum BDNF levels and HAMD-24 scores at D28 post treatment were also negatively correlated (*R*^2^ = 0.11, *p* = 0.03).

**FIGURE 4:**
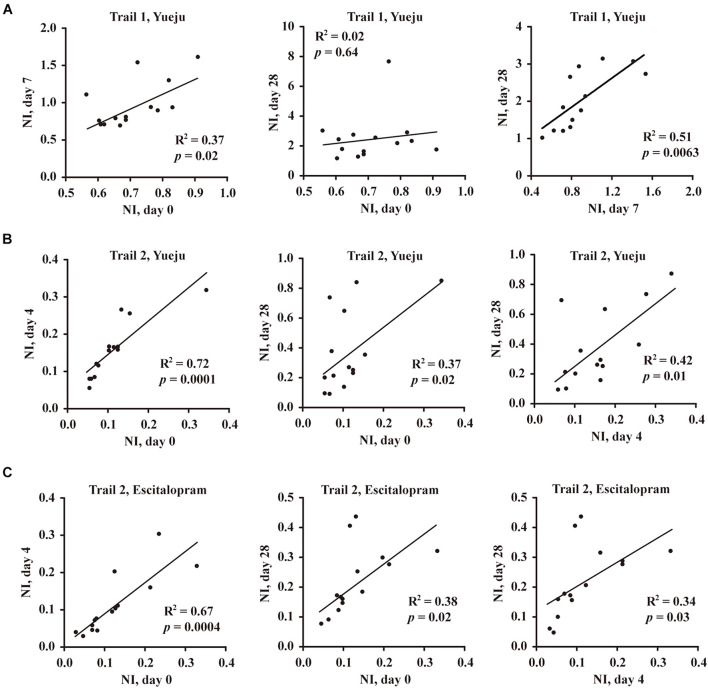
Correlation analysis of neuroplasticity index (NI), the ratio of BDNF to HAMD-24 score in Trial 1 and Trial 2. **(A)** Correlation of NI between baseline (Day 0, D0), early treatment time (D7), and final treatment time (D28) after Yueju pill treatment in Trial 1. **(B)** Correlation of NI between baseline (D0), early treatment time (D4), and final treatment time (D28) after Yueju pill treatment in Trial 2. **(C)** Correlation of NI between baseline (D0), early treatment time (D4), and final treatment time (D28) after escitalopram treatment in Trial 2.

When the ratio of serum BDNF level and HAMD-24 score (BDNF/HAMD-24), or NI, was evaluated, the NI values on baseline D0 and on D7 post treatment of Yueju were significantly correlated in Trial 1 (*R*^2^ = 0.37, *p* = 0.02, Figure 4A, left panel). NIs on D7 and D28 were also correlated (*R*^2^ = 0.51, *p* = 0.0063, Figure 4A, right panel). Consistently, the correlation of NI at baseline D0 and NI on D4 was significant in Trial 2 for either Yueju (*R*^2^ = 0.72, *p* = 0.0001, Figure 4B, left panel) or escitalopram (*R*^2^ = 0.67, *p* = 0.0004, Figure 4C, left panel). Furthermore, there existed a significant correlation of NI on baseline D0 and on D28 for Yueju treatment (*R*^2^ = 0.37, *p* = 0.02, Figure 4B, middle panel) or escitalopram treatment (*R*^2^ = 0.38, *p* = 0.02, Figure 4C, middle panel) as well as correlations between NIs on D4 and on D28 for Yueju (*R*^2^ = 0.42, *p* = 0.01, Figure 4B, right panel) or escitalopram treatment (*R*^2^ = 0.34, *p* = 0.03, Figure 4C, right panel) in Trial 2.

**FIGURE 5:**
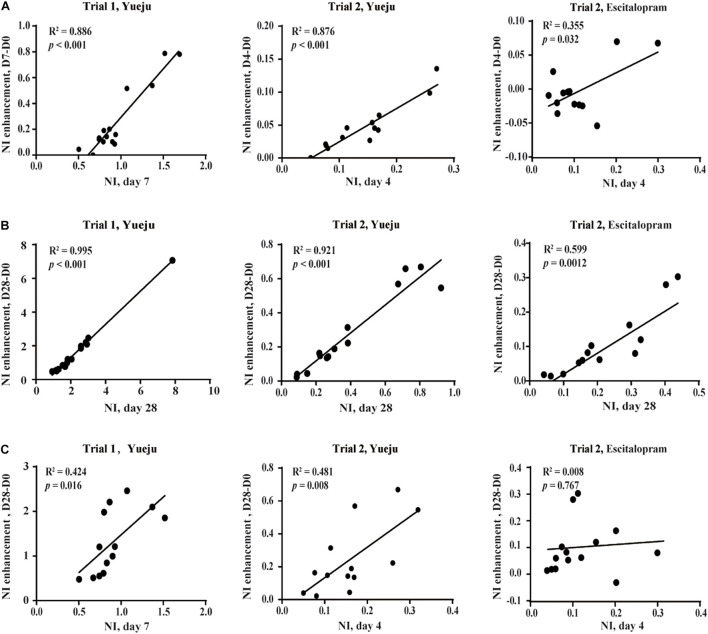
Correlation analysis of NI enhancement, the change of NI within a given period of time. **(A)** The correlation between early NI and early NI enhancement. **(B)** The correlation between final NI and final NI enhancement. **(C)** The correlation between early NI and final NI enhancement. The early NI was on D7 of Yueju treatment in Trial 1 and on D4 of Yueju or escitalopram in Trial 2. The early NI enhancement was the difference of NI between D7 and D0 in Trial 1 and between D4 and D0 in Trial 2. The final NI was on D28 and the final NI enhancement was the difference of NI between D28 and D0 in both trials.

On the basis of NI, a derivative measurement of NI enhancement was further defined: NI enhancement referred to the difference in NI values following a given time period of treatment. There was a significant correlation between the early NI (D7) and the early NI enhancement from D0 to D7 (NI_*D7*_–NI_*D0*_) in Trial 1 (*R*^2^ = 0.886, *p* < 0.001, Figure 5A, left panel), which was replicated in Trial 2 between the early NI (D4) and enhancement of NI from the D0 to D4 (NI_*D4*_–NI_*D0*_) for Yueju (*R*^2^ = 0.876, *p* < 0.001, Figure 5B, middle panel). This correlation was also significant for escitalopram treatment (*R*^2^ = 0.706, *p* = 0.0006, Figure 5A, right panel). Consistently, there was significant correlation between the final NI (D28) and final NI enhancement (NI_*D28*_–NI_*D0*_) for Yueju in Trial 1 (*R*^2^ = 0.995, *p* < 0.001, Figure 5B, left panel) and Trial 2 (*R*^2^ = 0.921, *p* < 0.001, Figure 5B, middle panel) and for escitalopram in Trial 2 (*R*^2^ = 0.599, *p* = 0.0012, Figure 5B, right panel). Furthermore, there was a significant correlation between the early NI (D7) and the final NI enhancement from D0 to D28 (NI_*D28*_–NI_*D0*_) in Trial 1 (*R*^2^ = 0.424, *p* = 0.016, Figure 5C, left panel), which was replicated in Trial 2 (*R*^2^ = 0.481, *p* = 0.008, Figure 5C, middle panel). However, there was no such correlation for escitalopram.

### Receiver Operating Characteristic Curve Analysis of Early Neuroplasticity Index Enhancement

**FIGURE 6:**
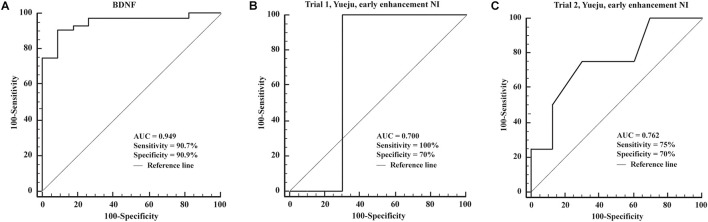
Receiver operating characteristic curves of the detection power of different markers. **(A)** ROC curves of the detection of BDNF. **(B,C)** ROC curves of the detection of the biomarker of early NI enhancement in Trial 1 and Trial 2.

Brain-derived neurotrophic factor levels were only used for ROC analysis of diagnosis of depression, as no correlation was found for treatment effect by it alone. The AUC (area under the ROC curve) for detection of depression using BDNF was 0.949, with a diagnostic sensitivity and specificity of 90.7 and 90.9% (Figure 6A). As the NI values showed good correlation between baseline, early time, final time or the enhancement from the baseline, we tested if they can be further used for ROC curve analysis to predict the long-term antidepressant response. Using 60% of reduction of HAMD-24 scores by 28 days of treatment as an efficacy index, we found that early NI enhancement showed considerable sensitivity and specificity for Yueju treatment prediction in Trial 1 and Trial 2 with all values of AUC (0.7 (Figures 6B,C). However, due to the insufficient number of subjects meeting the criteria of treatment efficacy, it was impossible to perform the ROC analysis using NI enhancement for escitalopram treatment.

## Discussion

In this study, we investigated whether the ratio of serum BDNF level and depression scale of HAMD-24, or NI, was useful for prediction of the long-term antidepressant outcome, using Yueju and/or escitalopram in two trials. In the preliminary open-labeled trial, we found both symptom alleviation and serum BDNF enhancement as early as 1 week post a conventional low dose of Yueju. We also revealed significant correlation between NIs at baseline and at the early treatment time. In the confirmatory random controlled double-blinded clinical trial, we found both escitalopram and Yueju pill resulted in early improvement of depression symptom, and comparable antidepressant outcome after 4 weeks of treatment. Unlike Yueju, escitalopram treatment failed to change BDNF levels overtime. However, baseline NI, early treatment NI and final treatment NI were all significantly correlated for both Yueju and escitalopram. Furthermore, the early NI was also correlated to the final enhancement of NI for Yueju in both trials. Finally, ROC analysis indicated the final antidepressant efficacy of Yueju treatment was predictable on the basis of early NI enhancement. The results suggest that the NI may represent an individual’s trait that links BDNF with depression symptom, and early change of NI may predict long-term antidepressant efficacy.

Brain-derived neurotrophic factor is a neurotrophin that has been linked to the viability of neurons in brain circuits for depression regulation (Molendijk et al., 2011), and it has been argued that BDNF is a key player for neural plasticity underlying depression and antidepressant activity (Warner-Schmidt and Duman, 2006). Several lines of studies demonstrated the reduced levels of BDNF in MDD patients (Dreimüller et al., 2012). Consistent with this, the present study showed lower serum BDNF levels in MDD patients. The serum BDNF levels of depression patients also were negatively correlated with the degree of depression, when the data from the two trials were collapsed. Furthermore, we found MDD can be reliably predicted with high sensitivity and specificity on the basis of serum BDNF levels. Despite the utility of BDNF for prediction of MDD, our data indicated that it was not useful for prediction of antidepressant efficacy (Dreimüller et al., 2012; Wilkinson et al., 2018). Unlike the increase of BDNF level post treatment of Yueju in both trials, BDNF levels were not changed in the escitalopram treatment group even escitalopram induced significant symptom improvement, consistent with a finding that escitalopram did not affect BDNF levels at either early and late time points (Matrisciano et al., 2009).

The recovery of neuroplasticity is considered to be an important neural mechanism for antidepressant activity (Castrén and Antila, 2017; Koopman and El Aidy, 2017; Franklin et al., 2018; Fox and Lobo, 2019). NI is used to characterize the relative contribution of neuroplasticity indicated by the BDNF level to depression symptoms indicated by the score of HAMD-24. The differences of NI in individuals may reflect different levels of sensitivity of depression symptoms to BDNF levels. NIs at the baseline, the early time of treatment and the final time of treatment were all correlated. Importantly, this correlation was found for both Yueju and escitalopram treatment, even though escitalopram group did not show increased BDNF levels or different doses of Yueju was used overtime. These suggest that NI may represent a trait constantly determining the role of BDNF in an individual’s depressive symptoms both at the disease baseline condition and following a certain antidepressant treatment. These intrinsic characteristics also led to the association between early or final treatment time NI with early or final NI enhancement, respectively. It is also noticeable that the measurement of HAMD-17 was not as sensitive as HAMD-24, which may indicate the importance of completeness of the depression symptom for NI. As HAMD-24 scaling and BDNF Elisa measurement are well developed techniques, NI can be reliably calculated and used in the clinical setting.

Partly due to the correlation of baseline NI with the NI at early or final treatment time, it was not surprising that early/final NI was significantly correlated with early/final NI enhancement for either Yueju or escitalopram treatment. This may contribute to the predictability with early NI enhancement to the final treatment efficacy of Yueju. The present study was unable to predict the treatment efficacy of escitalopram. This is technically ascribed to the insufficient number of subjects that met the criteria of treatment efficacy after 4 weeks of treatment of escitalopram. Other factors may also account for the difficulty: in contrast to the general increase of NI at both early and final treatment time of Yueju, NI was mostly reduced at the early treatment time, but augmented by the final treatment time of escitalopram. Consistently, we found the early treatment NI was correlated with the final NI enhancement for Yueju, but not for escitalopram. The definition of NI or related index may have some applications in testing other antidepressants. For example, ketamine is known for the neuroplasticity-dependent mechanism. For escitalopram or other 5-HT based antidepressants, we speculate to use the index like the ratio of the 5-HT improvement value to HAMD-24 score at somehow early stage, to predict the outcome from the contribution of early 5-HT response to behavioral improvement. Certainly, these warrant further investigations. These possibilities should be addressed in future studies by use of a variety of antidepressants for a longer treatment time with a larger number of MDD subjects.

In the present study, we also revealed that the clinical symptom improvement as early as 4 days after treatment of escitalopram. To the best of our knowledge, this finding was reported for the first time. It warrants further clinical and preclinical investigations as escitalopram has become one of the most frequently prescribed antidepressants for its relatively fast action among SSRIs (Pastoor and Gobburu, 2014). Consistent with the finding on combination of the high dose of Yueju pill with fluoxetine achieved early symptom improvement, the present study provided evidence directly showing this dose of Yueju pill by itself was capable to elicit early-onset antidepressant effects in patients (Wu et al., 2015). It appeared there was a larger attenuation of the depressive symptoms by Yueju, compared to escitalopram, although it did not reach the statistic significance. These are in agreement with the findings using different animal models that displayed an immediate and lasting antidepressant effect following a single high dose of Yueju pill (Tang et al., 2015). Additionally, the present study also showed the long-term antidepressant efficacy of conventional low dose of Yueju in both trials. As low dose of Yueju has been used safely over 800 years clinically, it may be useful for a long-term treatment that many times is required for prevention of recurrence of the disorder.

## Limitations and Conclusion

In conclusion, we demonstrate that NI characterized the role of neuroplasticity in antidepressant treatment. The change of the NI after a short period time of treatment may indicate how the neural plasticity contributes to symptom improvement, which is helpful for the prediction of long-term antidepressant efficacy. Whether this measurement can be generalized to other antidepressant treatment remains to be determined. Nonetheless, the present study indicates the putative composite biomarker at the early time to predict the long-term antidepressant response, which offers novel insights to develop effective biomarker for antidepressant treatment efficacy to improve the treatment outcomes.

## Data Availability Statement

The raw data supporting the conclusions of this article will be made available by the authors, without undue reservation.

## Ethics Statement

The studies involving human participants were reviewed and approved by the Institutional Review Board of The Fourth People’s Hospital of Taizhou, Jiangsu Province. The patients/participants provided their written informed consent to participate in this study. Written informed consent was obtained from the individual(s) for the publication of any potentially identifiable images or data included in this article.

## Author Contributions

YZ, BC, TW, YL, ZC, ZZ, JM, XZ, YY, HW, and GC performed the experiments and analyzed the data. YZ and GC conceived the experiments and contributed to the interpretation and to writing the manuscript. All authors revised the manuscript.

## Conflict of Interest

The authors declare that the research was conducted in the absence of any commercial or financial relationships that could be construed as a potential conflict of interest.

## Publisher’s Note

All claims expressed in this article are solely those of the authors and do not necessarily represent those of their affiliated organizations, or those of the publisher, the editors and the reviewers. Any product that may be evaluated in this article, or claim that may be made by its manufacturer, is not guaranteed or endorsed by the publisher.
